# Ontogenetic resource-use strategies in a rare long-lived cycad along environmental gradients

**DOI:** 10.1093/conphys/cou034

**Published:** 2014-08-20

**Authors:** Juan C. Álvarez-Yépiz, Alejandro Cueva, Martin Dovčiak, Mark Teece, Enrico A. Yepez

**Affiliations:** 1 Department of Environmental and Forest Biology, State University of New York College of Environmental Science and Forestry, Syracuse, NY 13210, USA; 2 Departamento de Biología de la Conservación, Centro de Investigación Científica y de Educación Superior de Ensenada, Baja California 22860, México; 3 Department of Chemistry, State University of New York College of Environmental Science and Forestry, Syracuse, NY 13210, USA; 4 Departamento de Ciencias del Agua y Medio Ambiente, Instituto Tecnológico de Sonora, Ciudad Obregón, Sonora 85000, México

**Keywords:** Adult niche, biological nitrogen fixation, drought stress, facilitation, regeneration niche, resource-use efficiency

## Abstract

Functional traits can drive plant responses to short- and long-term stressful conditions, with potential effects on species persistence in local habitats, changes in population size and structure, and potential species range shifts in changing environments. We investigated whether ecophysiological traits in a rare cycad vary along environmental gradients and with ontogeny to understand intra-specific resource-use variation (e.g. symbiotic nitrogen fixation, nitrogen- and water-use efficiency) and possible species adaptations for different environments. Environmental gradients were characterized with 14 soil and topographic variables. Nitrogen- and water-use efficiency improved with ontogeny (from seedling to juvenile and adult stages) but declined as soil fertility decreased with increasing elevation. Conversely, reliance on symbiotic nitrogen fixation increased with elevation and varied slightly with ontogeny. Improved water-use efficiency at lower elevation and nitrogen fixation at higher elevation may represent key functional strategies for maintaining the lower and upper altitudinal species range limits, especially in arid environments where stressful conditions are intensifying due to climatic and land-use changes. In addition to facilitation linked to the regeneration niche, improved resource-use efficiency linked to the adult niche may strongly influence cycad distribution and persistence in contemporary environments. A functional approach to conservation of rare or endangered plant species may be needed in order to target the most sensitive stages to changing environmental conditions and to better understand potential range shifts and adaptive responses to global land-use and climate changes.

## Abstract (precis)

We studied carbon and nitrogen acquisition and water-use efficiency across the ontogeny of a rare cycad in relation to environmental gradients. Increased water-use efficiency at lower drier elevations and nitrogen fixation at upper elevations with nutrient poor soils may help maintaining the lower and upper altitudinal species range limits

## Introduction

Functional traits can drive plant responses to short- and long-term stressful environmental conditions, such as water or nutrient stress, which represent two major environmental filters in arid environments (Chapin *et al.*, 1987; Condit *et al.*, 2013). Predictability of trait function in response to multiple stressors during species ontogeny can provide deeper insights into survival at sensitive early life stages (e.g. seedlings) and long-term species persistence in changing environments (Chapin *et al.*, 1993; Fonseca *et al.*, 2000; Cavender-Bares *et al.*, 2004). Functional traits related to resource acquisition (e.g. carbon assimilation) are often positively correlated with soil nutrient content, whereas the opposite relationship is usually observed for biological fixation of atmospheric nitrogen (Aerts and Chapin, 1999; Zahran, 1999; Bai *et al.*, 2009; Ordoñez *et al.*, 2009). In addition to variation along environmental gradients, leaf traits can also vary predictably with ontogeny. For example, photosynthetic rates on a leaf area basis and nitrogen fixation usually tend to increase with ontogeny (Donovan and Ehleringer, 1991; Fredericksen *et al.*, 1996; Cavender-Bares and Bazzaz, 2000; Ishida *et al.*, 2005). It is therefore expected that trait function should vary predictably along resource gradients and ontogeny, with potential effects on species performance and future species distribution.

Current gymnosperm (e.g. cycads, conifers) distribution is thought to be related to the contraction to refuge habitats due to past climatic changes and the poor competition with angiosperm species due to reproductive and physiological constraints (Bond, 1989; Becker, 2000; Preece *et al.*, 2007; Álvarez-Yépiz *et al.*, 2014). Ecophysiological traits can give us insights into plant responses to short- and long-term water stress, the most important limiting factor for plants in arid ecosystems. Functional short-term responses to water stress may include lower carbon assimilation and higher water-use efficiency, while longer-term responses may involve additional morphological adaptations, such as sclerophyllous leaves to improve resistance against water loss and physical damage (Chaves *et al.*, 2003, 2009). Leaf shedding in response to drought is an obvious adaptation of drought-deciduous species from arid environments, such as tropical dry forest systems (Bullock and Solis-Magallanes, 1990; Olivares and Medina, 1992). However, evergreen species from tropical dry forests, such as the long-lived, N_2_-fixer cycad *Dioon sonorense*, should have physiological adaptations or strategies to resist water stress while maintaining a positive carbon balance [e.g. higher water-use efficiency (WUE); Givnish, 2002; Nobel, 2009], which is especially important at the seedling stage.

Plant functional traits are usually studied at the regeneration niche because higher mortality rates during early ontogeny make this phase a critical period for selection of plant traits (Reich, 2000). During this phase, facilitation by canopy shading can provide protection and a less fluctuating microenvironment that may increase seedling survival and growth, especially in species from arid or other drought-prone environments, such as cacti, pines or cycads (Turner *et al.*, 1966; Valiente-Banuet and Ezcurra, 1991; Dovčiak *et al.*, 2005; Álvarez-Yépiz *et al.*, 2014). The regeneration niche, commonly defined by the environmental conditions in early plant ontogeny (Grubb, 1977), therefore seems vital for successful survival of early plant life stages. Nonetheless, gradual recruitment into more advanced juvenile and adult life stages is equally critical for species population persistence (e.g. Dovčiak *et al.*, 2001; Álvarez-Yépiz *et al.*, 2011). Species adaptations related to the adult niche should be particularly relevant for the long-term persistence and future distribution of long-lived species, such as many gymnosperms (including cycads), for which adult plants contribute the most to species population growth rates (Silvertown *et al.*, 1996; Raimondo and Donaldson, 2003; Álvarez-Yépiz *et al.*, 2011). Therefore, information on species resource-use strategies (e.g. water, carbon and nitrogen utilization) at different ontogenetic stages and in different environments may be particularly useful in order to implement better conservation practices, especially during extreme climatic events or increasing aridity over longer temporal scales, as projected by various climate-change scenarios (Easterling *et al.*, 2000; Walther *et al.*, 2002; Choat *et al.*, 2012; Dai, 2013).

Assuming that the quantification of species functional traits by life stage can help us to gain insights into intra-specific resource-use variation and possible species adaptations for different environments during their ontogeny, we specifically asked: does the lower resource use in seedlings and juveniles more strongly reflect an adaptation of the younger life stages to different environmental conditions or developmental constraints that prevent them from achieving more advantageous trait values? We expect carbon assimilation and nitrogen- and water-use efficiency to be positively related to soil fertility, while symbiotic nitrogen fixation should be related negatively to soil fertility. However, as ontogeny progresses plants should become more efficient (i.e. higher water-use efficiency, photosynthetic nitrogen-use efficiency and symbiotic nitrogen fixation) given the potential adaptive significance of these key functional strategies for long-lived gymnosperms inhabiting water- and nutrient-limited environments.

## Materials and methods

### Description of species and study area

**Figure 1:**
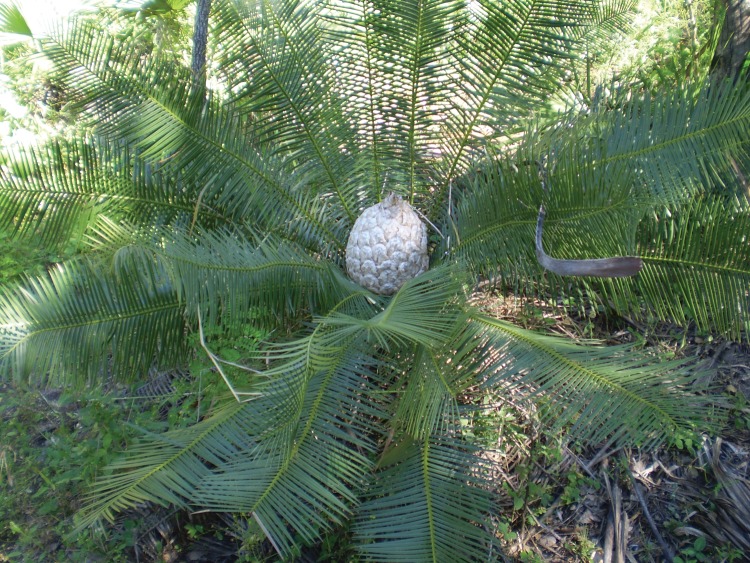
Female plant of the rare cycad *Dioon sonorense* in its natural habitat in northwestern Mexico. Photo credit: J.C.Á.-Y.

*Dioon sonorense* (De Luca, Sabato and Vazq.Torres) Chemnick and T.J. Greg. and Salas-Morales (Zamiaceae; previously known as *Dioon tomasellii* var. *sonorense*) is an endemic endangered cycad of northwestern Mexico (IUCN, 2012). *Dioon sonorense* is a dioecious understory species, with stems usually ≤2 m tall and lanceolate pinnate leaves usually ≤1 m long at maturity (Fig. 1). *Dioon* species (e.g. *Dioon edule* Lindl.) are known to grow very slowly, reaching considerable age (≥1000 years; Vovides, 1990). Most cycads appear to fix atmospheric nitrogen through symbiotic relationships with cyanobacteria within coralloid roots (Lindblad and Costa, 2002). The distribution of *Dioon sonorense* seems roughly restricted to 500–1100 m above sea level and reaches higher plant abundance at mid-range (Álvarez-Yépiz *et al.*, 2011). It has been suggested that the long-term persistence of *D. sonorense* is threatened by small adult population sizes, low-quality habitat and the combination of low fecundity and poor recruitment, as well as additional extrinsic factors, such as illegal extraction and land use change (Álvarez-Yépiz *et al.*, 2011). *Dioon sonorense* adults tend to occupy more open spaces, whereas seedlings tend to grow under a relatively permanent canopy cover (e.g. under evergreens). Rare, small populations are particularly vulnerable to climate extremes; for example, the heavy rains from hurricane Norbert in October 2008 washed down one of the populations (∼90% of plants) at higher elevation (J.C.Á.-Y., personal observation).

**Figure 2:**
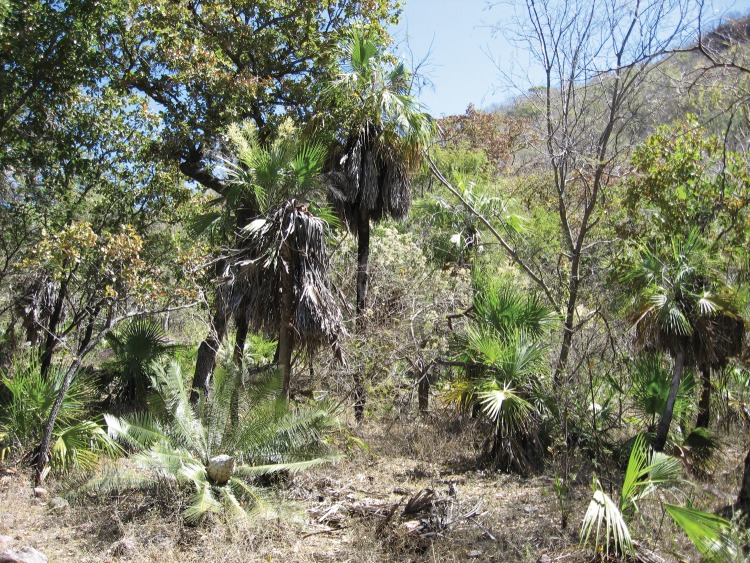
Habitat of the rare cycad *D. sonorense* in the ecotone between tropical dry and oak forests in northwestern Mexico. Photo credit: J.C.Á.-Y.

The study sites are located in the Sierra de Alamos-Rio Cuchujaqui Biosphere Reserve, a 93 000 ha protected area located in the southeastern part of the State of Sonora, Mexico (27°12′–26°53′ N, 109°03′–108°29′ W), where agroforestry is widespread and represents the main economic activity for local communities. The climate is warm and semi-arid, with a total annual precipitation of 712 mm and marked seasons; the dry season is from November to June and the wet season from June to October, when ∼80% of annual precipitation occurs (Álvarez-Yépiz *et al.*, 2008). The lowest temperatures can dip below freezing in winter, while the highest summer temperatures can be >40°C, with a mean annual temperature of 24.3°C (Comisión Nacional del Agua, San Bernardo Meteorological Station). Elevation in the Biosphere Reserve ranges from 300 to 1800 m above sea level, and shallow lithosol soils predominate in the mountainous portions of the Biosphere Reserve, where steep slopes prevail (INEGI, 1985). The three main vegetation types of the Biosphere Reserve (and of the sites where *D. sonorense* occurs) are tropical dry forest at low elevations, oak forest at mid-elevation (Fig. 2) and pine-oak forest at higher elevation.

### Leaf trait measurements

We collected a total of 81 leaf samples from the 27 study plots located along an elevation gradient (500–1000 m above sea level) where the nine study populations occur (i.e. three plots per population). We collected one composite leaf sample from three different leaflets from a randomly selected plant per life stage (seedling, juvenile and adult). Life-stage classification was based on individual height and reproductive characteristics (Álvarez-Yépiz *et al.*, 2011), as follows: seedlings, <50 cm tall; juveniles, ≥50 but <100 cm tall; and adults, ≥100 cm tall. Measurements of average net CO_2_ assimilation (*A*) and transpiration (*E*) rates were performed on the same 81 plants used for leaf tissue collection prior to collecting leaf samples. We obtained 10 readings (per plant per life stage) from three fully developed and exposed leaflets placed within the chamber. Gas-exchange measurements were conducted at ambient conditions of (chamber) temperature (34.02 ± 5.63°C), CO_2_ (391.27 ± 22.66 ppm) and photosynthetically active radiation (925.57 ± 483.81 photosynthetic photon flux density) between 10.00 and 12.00 h at the beginning of the rainy season (June/July) using an LCpro+ Portable Photosynthesis System (ADC BioScientific Ltd, Great Amwell, Hertfordshire, UK).

All leaf samples were analysed for weight percentage of nitrogen (*N*) and for δ^13^C and δ^15^N composition. Standard procedures were followed to process and analyse 2.5–3.0 mg of solid samples for carbon and nitrogen concentrations and carbon (δ^13^C) and nitrogen (δ^15^N) stable isotope composition using a Costech Elemental Analyzer coupled to a ThermoFinnigan Delta XL Plus stable isotope ratio mass spectrometer via ThermoFinnigan Conflo III interface (Teece *et al.*, 2011). The accuracy and precision of the stable isotope measurements (expressed in the standard per mille notation relative to Vienna Pee Dee Belemnite for δ^13^C and atmospheric nitrogen for δ^15^N) were verified using National Institute of Standards and Technology RM8573 [δ^13^C = −26.4 ± 0.1‰ (*n* = 38) and δ^15^N = −4.5 ± 0.3‰ (*n* = 38)], RM8574 [δ^13^C = +37.6 ± 0.2‰ (*n* = 38) and δ^15^N = +47.6 ± 0.3‰ (*n* = 38)]. Daily precision of the instrument was verified by repeated analyses of internal laboratory standards, including acetanilide [δ^13^C = −29.9 ± 0.1‰ and δ^15^N = −0.1 ± 0.4‰ (*n* = 9)], valine [δ^13^C = −10.9 ± 0.1‰, δ^15^N = −6.6 ± 0.3‰ (*n* = 5)] and daphnia [δ^13^C = −24.8 ± 0.1‰, δ^15^N = +17.2 ± 0.5‰ (*n* = 3)], during the sample runs. We also analysed one composite leaf sample at each plot from a species without the ability to fix atmospheric nitrogen (i.e. 27 plants sampled from the oak *Quercus chihuahuensis* Trel.) to be used as reference values for biological atmospheric nitrogen fixation estimates (Shearer and Kohl, 1986). We used the natural ^15^N abundance method to estimate the percentage of nitrogen fixation in *D. sonorense* (Shearer and Kohl, 1986), as follows: N_2_ fixation = (δ^15^N_(reference)_ − δ^15^N_(N-fixing)_/δ^15^N_(reference)_ − *B*) × 100, where δ^15^N_(reference)_ is the mean value of the *Q. chihuahuensis*, δ^15^N_(N-fixing)_ is the mean value of *D. sonorense* per life stage, and *B* represents the mean value for N_2_-fixing plants when grown totally depending on N_2_. In the absence of *B* values for *D. sonorense*, we used a common value for tree legumes (−2‰) to test the potential importance of extreme *B* values (Högberg, 1997; Freitas *et al.*, 2010). Our calculated variable of biological N_2_ fixation was highly (linearly) correlated with *D. sonorense* δ^15^N values (Pearson's *r* = −0.87); we therefore focused on interpreting potential trends of biological fixation using *D. sonorense* δ^15^N raw values instead of the calculated percentages because these are not always accurate and are thus highly debated (see Michener and Lajtha, 2007). In the present study, low or more negative δ^15^N values indicate that higher leaf nitrogen content was potentially derived from atmospheric nitrogen fixation as opposed to, for example, from soil nitrogen (Shearer and Kohl, 1986; Högberg, 1997). Likewise, δ^13^C raw values were used due their widely known (positive) relationship with long-term water-use efficiency (see Michener and Lajtha, 2007).

In summary, although our sample size was limited by the nature of our rare species, we obtained seven key ecophysiological traits related to carbon assimilation, water-use efficiency and nitrogen fixation in three different life stages occurring at nine different populations (81 sampled plants in total; 27 individuals per stage). The traits included in our analysis are as follows: (in micromoles of carbon dioxide per second per square metre); *E*, instantaneous transpiration rate (in millimoles of water per second per square metre); *N*, time-integrated leaf nitrogen concentration (expressed as a percentage); PNUE, the instantaneous photosynthetic nitrogen-use efficiency, the ratio of carbon assimilation rate per unit leaf nitrogen (in micromoles of carbon dioxide per second per mole of nitrogen; WUE, the instantaneous water-use efficiency, the ratio of *A*/*E* (in micromoles of carbon dioxide per millimole of water); and δ^13^C and δ^15^N, the time-integrated carbon and nitrogen stable isotope ratios (expressed per mille).

### Characterization of environmental gradients

**Figure 3:**
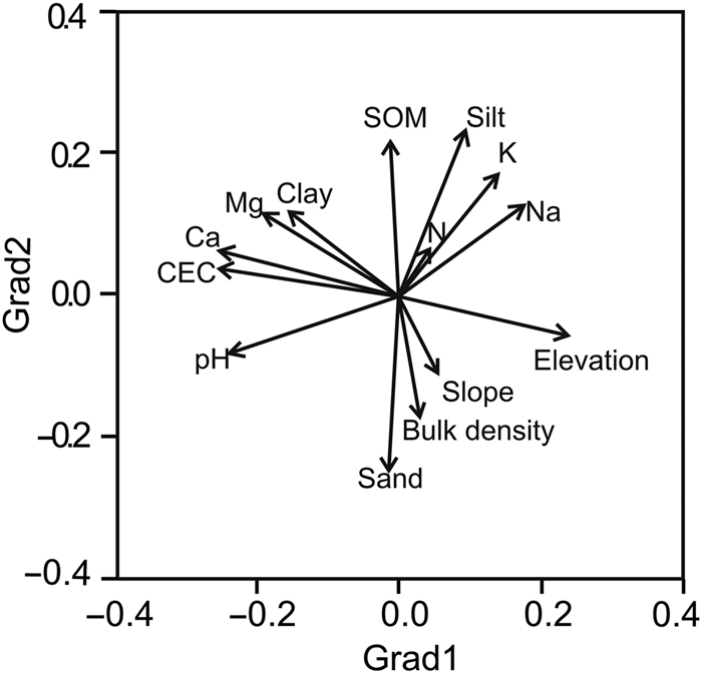
Ordination results of soil and topographic variables using principal component analysis. Principal component 1 (Grad1) and principal component 2 (Grad2) explained 29 and 24% of the variance, respectively (53% total explained variance). Full names of environmental variables are given in the Materials and methods section.

Plot locations and elevations were obtained using a GPS (MAP 76S; Garmin) and confirmed with topographic maps. We recorded steepness of the slope at each plot using a clinometer. We sampled soils by stratifying each plot into upper, middle and lower slope sections. We collected nine soil samples per plot (three from each stratum) from a depth of 0–30 cm (soil core of 10 cm in diameter) and combined them into one composite soil sample per plot. Soils were characterized by measuring 12 physical and chemical soil variables using methods from Richard (1964). Soil organic matter content (SOM) was calculated using the Walkley–Black method, total nitrogen (*N*) using the Kjeldahl method, bulk density using the paraffin method, and soil pH was determined with a potentiometer. The content of clay, silt and sand (texture) was determined by the Bouyoucos method. Cation exchange capacity (CEC) and cation concentrations (Mg, Na, K and Ca) were estimated by the ammonium acetate method. The data set with two topographical variables and 12 soil variables for each of the 27 plots was used to run a principal component analysis (PCA) to simplify the correlation structure among variables and extract the main environmental gradients (principal component scores) to be used in subsequent analysis (Table 1 and Fig. 3). We used scree tests to select the number of principal components to be retained. In this case, the first and second principal components were retained and explained 29 and 24% of the variance, respectively (53% of cumulative variance). The first and most important component (hereafter, Grad1) represents a gradient of decreasing soil fertility with increasing elevation (where soil texture becomes coarser). The second component (hereafter, Grad2) represents a gradient of increasing soil fertility with decreasing slope steepness (where soil texture becomes finer; Fig. 3).

**Table 1:** Summary of topographic/edaphic variables used to obtain the environmental gradients

Variable/site	1	2	3	4	5	6	7	8	9
Bulk density (g cm^−3^)	1.85 (0.01)	1.78 (0.17)	1.93 (0.18)	1.23 (0.11)	1.55 (0.10)	1.82 (0.16)	1.92 (0.07)	1.71 (0.28)	1.79 (0.09)
Calcium (cmol kg^−1^)	3.60 (0.46)	1.93 (0.37)	2.53 (0.13)	2.40 (0.23)	0.80 (0.00)	2.60 (0.10)	1.07 (0.48)	2.13 (0.58)	3.40 (0.64)
CEC (cmol kg^−1^)	29.2 (1.67)	12.4 (0.65)	10.1 (0.09)	35.5 (0.61)	12.7 (0.13)	19.8 (2.31)	12.3 (0.29)	13.8 (0.75)	32.5 (2.48)
Clay (%)	25.9 (1.15)	23.3 (1.52)	27.2 (0.73)	33.9 (1.29)	27.7 (0.12)	26.1 (1.45)	26.7 (1.07)	22.9 (1.28)	31.2 (0.62)
Elevation (m)	622 (1.50)	800 (0.00)	857 (0.00)	500 (0.00)	800 (4.10)	720 (0.00)	998 (5.50)	960 (24.3)	670 (9.00)
Magnesium (cmol kg^−1^)	2.00 (0.23)	1.67 (0.18)	1.47 (0.13)	1.73 (0.13)	0.93 (0.13)	1.40 (0.10)	1.20 (0.23)	1.20 (0.23)	2.07 (0.18)
Organic matter (%)	2.78 (0.29)	4.55 (0.39)	4.52 (0.81)	6.14 (0.88)	2.82 (0.46)	4.50 (0.39)	4.42 (0.70)	5.15 (1.21)	2.69 (0.23)
pH	6.88 (0.05)	6.15 (0.14)	6.54 (0.23)	6.04 (0.09)	5.39 (0.22)	6.04 (0.10)	5.05 (0.13)	6.28 (0.40)	6.77 (0.23)
Potassium (cmol kg^−1^)	0.20 (0.00)	0.20 (0.00)	0.47 (0.13)	0.33 (0.07)	0.40 (0.12)	0.43 (0.10)	0.40 (0.12)	0.27 (0.07)	0.33 (0.13)
Sand (%)	56.9 (2.94)	46.9 (3.02)	48.9 (0.67)	33.8 (0.43)	42.3 (2.11)	56.2 (2.89)	40.7 (2.82)	58.4 (3.14)	50.8 (5.84)
Silt (%)	17.1 (4.08)	29.8 (1.49)	23.9 (1.26)	32.3 (1.71)	29.9 (2.02)	17.6 (1.45)	32.6 (1.94)	18.7 (3.09)	18.0 (5.29)
Slope (°)	48.3 (1.67)	33.7 (1.86)	10.0 (0.00)	25.0 (2.89)	51.7 (1.67)	8.3 (0.88)	36.7 (6.67)	36.6 (6.67)	33.3 (12.0)
Sodium (cmol kg^−1^)	0.20 (0.00)	0.20 (0.00)	0.20 (0.00)	0.20 (0.00)	0.20 (0.00)	0.20 (0.00)	0.20 (0.00)	0.33 (0.07)	0.2 (0.00)
Total nitrogen (%)	0.15 (0.01)	0.26 (0.02)	0.24 (0.06)	0.27 (0.10)	0.09 (0.02)	0.26 (0.02)	0.32 (0.07)	0.31 (0.05)	0.17 (0.04)

Values are means (±SEM); *n* = 3 samples per site (studied population). Abbreviation: CEC, cation exchange capacity. See Materials and methods section for additional details.

### Data analysis

We compared each of the functional traits among life stages using one-way analysis of variance (ANOVA) followed by Tukey's HSD tests. We performed stepwise analysis of covariance (ANCOVA) models with each functional trait as a response variable, life stage as a factor and the environmental gradients (Grad1 and Grad2) as covariates. A weighted least-squares model was performed between each response variable and the parameters that minimized the Akaike's Information Criteria score in the stepwise model. We used the relative abundance of each life stage per plot as weights for each of the models to account for life-stage variation. Given that the order of factor levels (i.e. adult, juvenile and seedling life stages) could potentially affect the ANCOVA outcome, we altered the order of life stages (e.g. seedling, juvenile and adult), and similar statistical significance was obtained for adult and seedling parameters. Juvenile stage remained in the middle in both model runs; this makes biological sense because juveniles are an intermediate stage in life history of the species. Response variable transformation was performed (i.e. Log10) to achieve variance homogeneity and normality of model residuals in instantaneous measurements of *A*, *E* and WUE. In all statistical tests, we set the significance level to be α = 0.05. Finally, a PCA was performed to determine the nature and number of relevant axes of functional resource-use differentiation among life stages. All analyses were run in R (R Development Core Team, 2009).

## Results

Except for transpiration rate and leaf *N* concentration, trait values related to carbon assimilation and water-use efficiency increased with life stage (Table 2). Photosynthetic rate and photosynthetic nitrogen-use efficiency (*A* and PNUE), instantaneous water-use efficiency (WUE), δ^13^C (positively related to long-term WUE) increased, whereas transpiration rate and leaf *N* concentration (*E* and *N*) decreased with ontogeny (Table 2). Values of δ^15^N (negatively correlated with biological nitrogen fixation) showed an upward trend with ontogeny, but differences among life stages were not significant (*P* > 0.05). Interestingly, although leaf *N* was the highest in seedlings, their carbon assimilation rate was the lowest and their transpiration rate the highest (Table 2); consequently, seedlings had the lowest water-use and photosynthetic nitrogen-use efficiency (Table 2), which suggests that compared with adults, seedlings can be more negatively affected by seasonal drought as inferred from WUE and δ^13^C values, respectively.

**Table 2:** Functional traits; mean values per life stage in a rare cycad

Trait/stage	Seedling	Juvenile	Adult
*A*	5.56 (0.87)^a^	9.80 (0.94)^ab^	11.1 (0.56)^b^
*E*	8.00 (0.66)^a^	5.27 (0.43)^b^	4.77 (0.41)^b^
*N*	1.68 (0.09)^a^	1.41 (0.08)^b^	1.47 (0.08)^b^
PNUE	32.5 (5.00)^a^	72.08 (7.81)^b^	82.2 (6.69)^b^
WUE	1.07 (0.25)^a^	2.20 (0.28)^b^	2.73 (0.23)^b^
δ^13^C	−27.15 (0.23)^a^	−26.58 (0.17)^ab^	−25.93 (0.19)^b^
δ^15^N	−1.02 (0.13)	−1.12 (0.19)	−1.37 (0.12)

Values are means (±SEM); *n* = 27 samples per life stage. Different letters indicate significant differences with Tukey's HSD test (*P* ≤ 0.01 in all significant comparisons except for PNUE juvenile–seedling with *P* = 0.02). Abbreviations: *A*, instantaneous photosynthetic rate (in micromoles of carbon dioxide per second per square metre); *E*, instantaneous transpiration rate (in millimoles of water per second per square metre); *N*, leaf nitrogen concentration (expressed as a percentage); PNUE, instantaneous photosynthetic nitrogen-use efficiency (in micromoles of carbon dioxide per second per mole of nitrogen; WUE, instantaneous water-use efficiency (in micromoles of carbon dioxide per millimole of water); and δ^13^C and δ^15^N, stable carbon and nitrogen isotope ratios (expressed as per mille).

**Figure 4:**
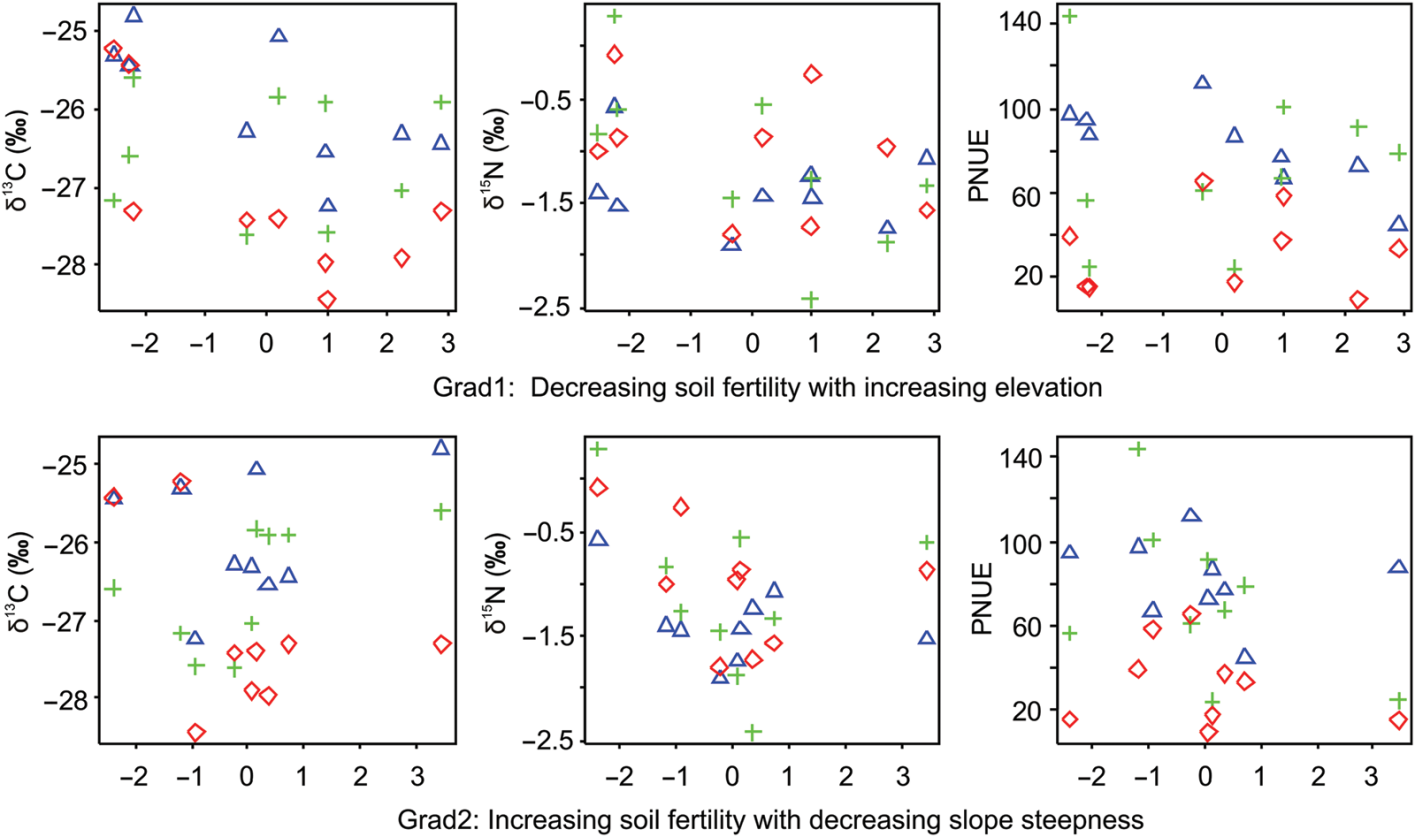
Relationships between key leaf traits related to long-term resource-use efficiency in the rare cycad *D. sonorense* and the main measured environmental gradients. Symbols represent mean values per life stage in the nine studied populations, as follows: triangle, adult; cross, juvenile; and rhomboid, seedling stage. Abbreviations: δ^13^C, carbon stable isotope ratio, used to infer long-term water-use efficiency; δ^15^N, nitrogen stable isotope ratio, used to infer reliance on symbiotic nitrogen fixation; and PNUE, photosynthetic nitrogen-use efficiency.

Trait variation along environmental gradients also showed some interesting trends. While *A* and *N* increased, PNUE, δ^13^C and δ^15^N decreased along the main environmental gradient, Grad1, i.e. decreasing soil fertility with increasing elevation (Table 3). On the contrary, instantaneous transpiration rate (*E*) and long-term water use efficiency (δ^13^C values) increased with Grad2, i.e. increasing slope steepness and coarse-texture soils (Table 3 and Figs 3 and 4). However, Grad1 and 2 were significant only for time-integrated traits (leaf *N*, δ^13^C and δ^15^N, *P* < 0.05; Table 3; see also Fig. 4). Likewise, the adult and seedling stages were significant in most ANCOVA models (*P* < 0.05; Table 3), which possibly suggests a greater importance of the measured traits for the adult and regeneration niches.

**Table 3:** Analysis of covariance of traits per life stage along environmental gradients

Trait^a^	Adult	Juvenile	Seedling	Grad1	Grad2	*r*^2^	*F*	*P*-value
*A*	***1.04***	** − 0.24**	** − *0.42***	0.02		0.20	6.24	<0.001
*E*	***0.65***	0.01	***0.21***		** − **0.02	0.15	4.45	0.006
*N*	***0.15***	** − **0.01	**0.09**	**0.01**		0.15	4.53	0.005
PNUE	***0.88***	** − 0.20**	** − *0.48***	** − **0.03		0.25	8.49	<0.001
WUE	***0.38***	** − 0.22**	** − *0.59***			0.22	10.9	<0.001
δ^13^C	** − *25.87***	** − *0.764***	** − *1.188***	** − *0.210***	**0.128**	0.40	12.4	<0.001
δ^15^N	** − *1.41***	0.28	0.37	** − *0.13***		0.15	4.52	0.005

Selection of model parameters was based on Akaike's Information Criteria. *F*_3,77_ for *A*, *E*, *N* and PNUE, and *F*_2,78_ for WUE. ^a^Except for δ^13^C and δ^15^N, response variables are on a logarithmic scale. Bold numbers indicate parameters significant at *P* < 0.05; italics indicate parameters significant at *P* < 0.001. Grad1 represents a gradient of decreasing soil fertility with increasing elevation. Grad2 represents a gradient of increasing soil fertility with decreasing slope steepness. Full names and units of traits are given in the footnote to Table 2.

**Figure 5:**
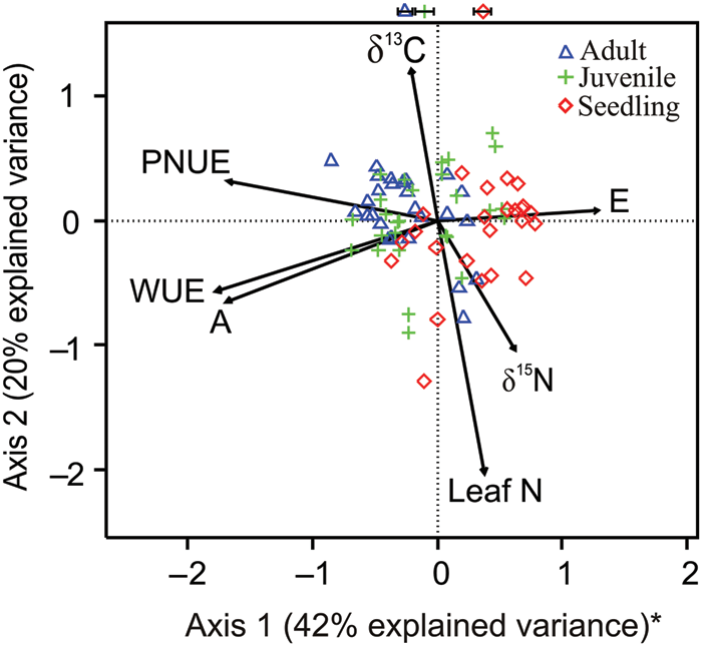
Ordination of functional traits of adults (triangles), juveniles (crosses) and seedlings (rhomboids) using principal component analysis. The combined explained variance of axes 1 and 2 was 62%. *Adults and juveniles were significantly separated from seedling plants along axis 1 using Welch's *t*-tests (*P* < 0.05). The position of symbols at the top of the figure indicates mean score values (±SEM, *n* = 27) of each life stage along axis 1. Full names of traits are given in Table 2.

The multivariate analysis shows the co-ordination of traits among the measured variables and provides additional support to our previous analyses separating ontogenetic stages along resource-use axes (Fig. 5). The first axis (42% of explained variance) shows the main physiological trade-off in arid environments between maximizing carbon gain (*A*) and minimizing water loss by transpiration (*E*). Water-use efficiency increased with ontogeny due to the higher photosynthetic rates and lower transpiration rates of adults and juveniles. The three life stages were differentiated by their predominant resource-use strategy along axis 1 (PNUE and WUE). Adults and seedlings showed the greatest separation (Fig. 5; *t* = −1.65, d.f. = 51, *P* < 0.001), followed by juveniles and seedlings (Fig. 5; *t* = −4.66, d.f. = 52, *P* < 0.001). Adults and juveniles showed no significant differentiation along axis 1 (Fig. 5; *t* = −1.65, d.f. = 51, *P* = 0.10). This distinction of life stages in the ordination space suggests that the first axis represents a resource-use strategy where adults (at the extreme left of Fig. 5) use water and nitrogen more efficiently than seedlings (at the extreme right of Fig. 5). The resource-use strategy of juveniles was somewhat intermediate but more similar to adults than to seedlings (Fig. 5). Axis 2 (additional 20% of explained variance) showed no significant separation among life stages, and thus interpretation of this resource axis seems more appropriate for the entire species. Axis 2 suggests a trade-off between increasing reliance on biological nitrogen fixation (in infertile soils at higher elevations; Table 3 and Figs 4 and 5) and increasing long-term WUE inferred from δ^13^C values (in more fertile soils at lower elevations; Table 3 and Figs 4 and 5).

## Discussion

We present, for the first time, several key time-integrated and *in situ* instantaneous measurements of leaf traits in the rare and endangered cycad, *D. sonorense*. Our trait data from nine populations are representative of the different habitats and elevations where *D. sonorense* occurs, and thus our analysis considerably improves our understanding of the main functional strategies in our study species and, potentially, in cycads from arid environments in general.

We initially asked whether the lower resource use in seedlings and juveniles more strongly reflected an adaptation of the younger life stages to different environmental conditions or a developmental constraint preventing them from achieving more advantageous trait values. Our results provide more evidence for the latter because resource use (mainly nitrogen and water) was consistently more efficient in more advanced ontogenetic stages. The long lifespan of *D. sonorense* and other cycads, such as *D. edule*, suggests that a higher resource-use efficiency (e.g. nitrogen, water and carbon) in adult plants should be particularly favoured and relevant for their persistence in arid ecosystems given their considerable longevity (e.g. on the scale of hundreds of years in *D. edule*; Vovides, 1990) and the greater importance of adults for future population growth rates (Raimondo and Donaldson, 2003; Álvarez-Yépiz *et al.*, 2011). Our data also suggest that seedlings may possibly be adapted to less extreme environmental conditions (e.g. light, temperature and drought stress levels) compared with juveniles and adults, which produce more resistant leaves with a well-developed waxy coating that reduces water loss (i.e. sclerophyllous leaves). This morphological adaptation seems to develop fully in *D. sonorense* seedlings after ∼60 days of germination when their leaves reach maturity (Álvarez-Yépiz *et al.*, 2014; O. Lopez-Bujanda *et al.*, unpublished data). Seedlings from another cycad species from dry environments (*D.* *edule*) showed a higher probability of dehydration during intense drought when their sclerophyllous leaves were not fully developed (Yáñez-Espinosa *et al.*, 2014). These results combined suggest a morpho-physiological threshold above which cycad seedling mortality due to dehydration should be comparatively lower.

**Figure 6:**
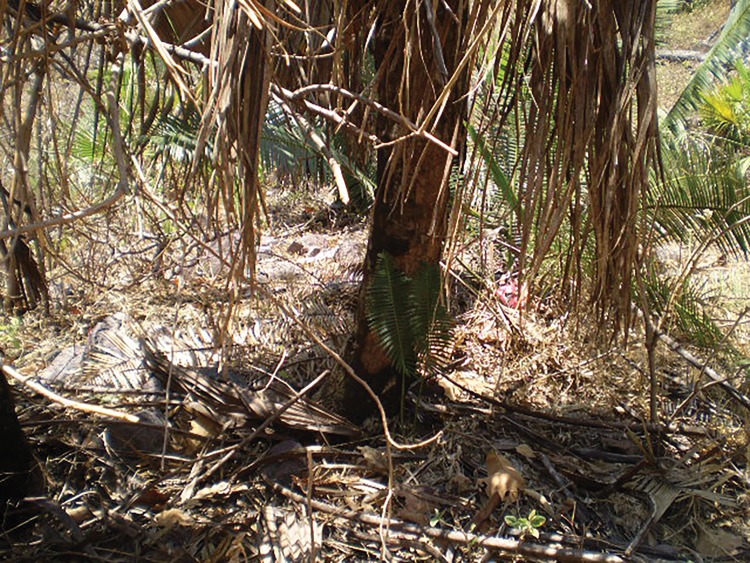
Seedlings from the rare cycad *D. sonorense* under the canopy of the endemic evergreen palm *Brahea aculeata* in their natural habitat during the dry season in northwestern Mexico. Photo credit: J.C.Á.-Y.

Cycad seedlings in field conditions in arid or semi-arid environments seem highly vulnerable to physical stress (e.g. due to drought), which is probably related to their high annual mortality rates (>40% in *D. sonorense*; Álvarez-Yépiz *et al.*, 2011, 2014; Yáñez-Espinosa *et al.*, 2014). Therefore, strategies to reduce water loss and thus mortality by desiccation (possibly due to hydraulic failure) may be critical for seedling survival in arid environments and future recruitment into the juvenile and adult stages (McDowell *et al.*, 2008; Nobel, 2009). For example, the negative effects of the underdeveloped sclerophyllous leaves of seedlings coupled with their lower resource-use efficiency (e.g. WUE) could be partly offset by the positive effects of tree canopy shading from conspecifics or heterospecific neighbours that ameliorates extreme microclimatic conditions (i.e. facilitation; Álvarez-Yépiz *et al.*, 2014; see Fig. 6). Facilitation by modern plant species was previously related to the persistence of plant species from Tertiary lineages (Valiente-Banuet *et al.*, 2006), but has only recently been specifically related to an increase in cycad seedling survival and growth rates by reducing competition and stress levels (see Álvarez-Yépiz *et al.*, 2014). Our data suggest that one of the important physiological mechanisms underlying facilitation in *D. sonorense* (and possibly in other cycads from arid environments) could be related to a reduction in seedling transpiration rates. Facilitation may then increase resource-use efficiency (i.e. WUE and PNUE) and therefore promote higher seedling survival and faster growth rates (Fig. 6; Álvarez-Yépiz *et al.*, 2014). Although we did not study cycad responses to frost or very low temperatures, these are likely to influence plant–plant interactions and cycad distributional patterns and they should be considered in future studies. Previous gas-exchange measurements from a limited sample in the largest and only viable *D. sonorense* population (in a relatively more fertile site) suggested an ontogenetic increase in carbon assimilation rates (see Álvarez-Yépiz *et al.*, 2011, 2014). Our more extensive ecophysiological trait data from nine populations located in different habitats suggest that cycad seedlings may be highly vulnerable to drought due their high transpiration and low carbon assimilation rates, whereas the opposite seems true for juvenile and adult plants with higher WUE (see Jordan and Nobel, 1981; Nobel, 2009 for similar results on cacti). Facilitation (in the regeneration stage) and resource-use efficiency (in the adult stage) may represent two overlooked explanations for cycad survival and persistence in contemporary arid environments; and they may provide new insights for conservation or management of cycads and other threatened plant species in stressful environments.

Elucidating plant functional responses to different levels of resource availability and environmental factors can provide insights regarding plant strategies to resist multiple stress factors (Chapin *et al.*, 1993; Grime, 2001). Two main physiological stressors of plant species in arid environments are related to the availability of water and soil nutrients (Chapin *et al.*, 1987; Condit *et al.*, 2013). Our soil data suggest that finer-textured soils prevail at lower elevations (and at gentler slopes) as opposed to coarser-textured soils at higher elevations (and at steeper slopes). Consistent with our data, fine-textured soils contain higher organic matter, nitrogen, water-holding capacity and availability of ions such as potassium, sodium, magnesium or calcium (Brady and Weil, 2007). Deficiency of any of these ions could have detrimental effects in plants because they are essential for key cell osmotic functions. For example, several studies have shown that potassium deficiency may affect the ability of plants to survive short- and long-term periods of water stress (Cakmak, 2005; Egilla *et al.*, 2005). Abundance of *D. sonorense* adult plants has been related to soil potassium content and slope steepness (Álvarez-Yépiz *et al.*, 2011). Therefore, our environmental and leaf trait data suggest that plant functional responses to water stress can be highly sensitive to topographical location, which affects soil fertility and moisture levels, as inferred with δ^13^C values.

Acquisition of resources, such carbon and water, seems generally to be positively related to soil fertility, whereas the opposite pattern should be expected for biological nitrogen fixation (Aerts and Chapin, 1999; Zahran, 1999; Bai *et al.*, 2009; Ordoñez *et al.*, 2009). In our study, nitrogen- and water-use efficiency (i.e. PNUE and δ^13^C values positively correlated with long-term WUE) increased at lower elevations with more fertile soils, whereas reliance on biological nitrogen fixation (inferred from δ^15^N values) increased at higher elevations with less fertile soils. In contrast to humid regions, drought stress in our study system can systematically increase leaf δ^13^C values because of higher temperatures and potential water loss at lower elevations where drought-deciduous species predominate (Brodribb and Hill, 1998; Van de Water *et al.*, 2002; Adams and Kolb, 2004; Álvarez-Yépiz *et al.*, 2008). Furthermore, we found average leaf δ^13^C cycad values close to 25‰ at lower elevations, which are typical of plants under water stress (Chapin *et al.*, 2002). In long-lived gymnosperms from arid environments, such as many cycads, water-use efficiency should probably increase in the drought-stressed environments and atmospheric nitrogen fixation in the nutrient-stressed environments. Failure to maintain this functional trade-off, for example at low/high elevations, may shrink our study species range; particularly, the lower and upper altitudinal limits in response to rapid land-use or climate change (see Easterling *et al.*, 2000; Davies and Shaw, 2001; Walther *et al.*, 2002).

### Conclusions

In this study, we quantified ecophysiological traits related to carbon and nitrogen acquisition and water-use efficiency across the ontogeny of a rare evergreen long-lived cycad species and in relationship to environmental gradients. Our trait data suggest that increased water-use efficiency at drier low elevations and nitrogen fixation at upper elevations with nutrient-poor soils may represent important functional strategies for maintaining the lower and upper altitudinal species range limits, especially in semi-arid environments during increasingly stressful conditions due to the ongoing climatic and land-use changes. Currently, the two most popular explanations for the distribution of gymnosperms (including cycad species) in contemporary environments are the contraction to refuge habitats due to past climatic changes and the poor competition with angiosperm species due to reproductive and physiological constraints (Bond, 1989; Becker, 2000; Preece *et al.*, 2007; Álvarez-Yépiz *et al.*, 2011, 2014). In addition, we argue that facilitation linked to the regeneration niche and enhanced resource-use efficiency linked to the adult niche may strongly influence present and future cycad distribution and persistence, particularly in stressful arid environments. Demographic models usually suggest that adults contribute the most to future population growth rates in long-lived plant species, and thus conservation actions are mainly recommended at the adult stage (see Álvarez-Yépiz *et al.*, 2011). However, adult recruitment will be possible if seedlings survive drought, nutrient limitation and other environmental stresses. We therefore recommend the inclusion of a functional perspective into conservation actions targeting rare or endangered plant species to identify, first, the most sensitive stages to changing environmental conditions and, second, potential species range shifts and adaptive responses to global land-use and climate change.
